# Suicide risk in male incarcerated individuals in Spain: clinical, criminological and prison-related correlates

**DOI:** 10.1186/s40359-023-01315-y

**Published:** 2023-09-21

**Authors:** Ellen Vorstenbosch, Ariadna Rodríguez-Liron, Enric Vicens-Pons, Mireia Félez-Nóbrega, Gemma Escuder-Romeva

**Affiliations:** 1 Parc Sanitari Sant Joan de Déu, Research, Teaching and Innovation Unit, C/ Dr. Antoni Pujadas 42, 08830 Sant Boi de Llobregat, Spain; 2 Instituto de Salud Carlos III, Centre for Biomedical Research On Mental Health (CIBERSAM), C/ Monforte de Lemos 3-5, 28029 Madrid, Spain; 3 Parc Sanitari Sant Joan de Déu, Penitentiary Psychiatric Hospitalization Unit of Catalonia, Carretera de Martorell a Capellades, Km 23, 08635 Sant Esteve Sesrovires, Spain

**Keywords:** Suicide risk, Correlates, Incarcerated individuals, Prison, Prevention program

## Abstract

**Background:**

Prison suicide is a complex phenomenon that may be influenced by individual, clinical, social and environmental factors. In Spain, few studies have explored the relationship with institutional, prison-related variables. The aim of this study is to examine correlates of suicide in a sample of male incarcerated individuals from 5 Spanish penitentiary centers.

**Methods:**

This present study entails a secondary data analysis, using data from the *Prevalence of mental disorders in prisons* study. This is a cross-sectional multicenter study conducted in 2007–2008 across 5 penitentiary centers in Spain. The Spanish version of the Plutchik suicide risk scale was used to assess the risk of suicide (those scoring ≥ 6 were considered to be at risk of suicide). Sociodemographic, clinical, criminological and prison-related data were collected via face-to face interviews and criminological data were confirmed using penitentiary records.

**Results:**

The final sample included 707 male incarcerated individuals (mean age 36.79 years ± 9.90 years). Several significant correlates associated with higher risk of suicide were identified including criminological factors (having committed a violent offense, being a recidivist), clinical factors (family history of mental disorders, the presence of mental disorders, having physical conditions, contact with a mental health specialist, medication treatment in the last 12 months), and prison-related determinants (workshop/training course participation) was significantly associated with lower suicide risk.

**Conclusions:**

Several correlates within a comprehensive range of sociodemographic, criminological, clinical and prison-related variables were identified. This information is primordial for preventing suicide and reducing the existing risk. The findings may contribute to developing effective suicide prevention programs within Spanish prison services. Importantly, future research must continue to investigate the nature of suicidal outcomes among incarcerated individuals.

**Supplementary Information:**

The online version contains supplementary material available at 10.1186/s40359-023-01315-y.

## Background

The prevalence of suicide and self-harm is higher in incarcerated people than in non-incarcerated people living in community. For instance, an international study conducted in 24 countries between 2011 and 2014 found that compared to individuals in the general population of the same sex and similar age, the rate ration of suicide in incarcerated individuals were typically higher than 3 in men and 9 in women [1]. Prison suicide is the most common cause of death in prison and represents a significant public health problem. According to the 2021 Annual Penal Statistics produced by the Council of Europe, prison suicide accounted for 34.8% of all causes of death in the Spanish prison population in 2020 [2]. For comparison, the European average is 28.4%, locating Spain among the countries with the highest rate (> 25% of the European median value) [2]_._ Studies on attempted suicide in Spanish prison populations found that 9% of the participants reported at least one attempt during incarceration, while 23% reported an attempt at some point in their lives [3, 4]. Moreover, in two Andalusian penitentiary centers (south of Spain), 34.2% of the incarcerated males reported suicidal thoughts and 33.5% could be considered at risk of suicide [4]. Studies from other countries show that an estimated 15 to 22% of the incarcerated individuals have a history of attempted suicide and 35 to 44% a history of suicidal ideation [5–8].

Prison suicide is a complex phenomenon that could be the result of an interaction between individual, clinical, social and environmental modifiable factors including prison environment [9, 10]. Indeed, a recent systematic review and meta-analysis including 35,351 suicides in 27 countries revealed that the main risk factors associated with suicide in prisons were clinical factors (including suicidal ideation during incarceration, a history of attempted suicide and current psychiatric diagnosis), criminological factors (such as remand status, serving a life sentence and being convicted of a violent offence) and institutional determinants (namely, occupation of a single cell and having no social visits) [11]. With respect to the latter, institutional or prison-related determinants are also associated with attempted suicide during incarceration. A recent meta-analysis comprising 19,882 individuals (including 6.5% women) from 20 studies covering 20 countries showed that the main prison-related risk factors were solitary confinement, victimization, and poor social support while incarcerated [12]. Others reported that incarcerated individuals residing in environments with greater deprivations such as a single cell or high secure wards, presented higher suicide risk [13–15]. On the other hand, a large international study examined several prison-level factors (including overcrowding, prisoner-staff ratio, prison population turnover ratios, and the management of prison health care), and found that most of these factors were not associated with prison suicide rates [1].

In Spain, some studies in the south or southeast explored the association between suicidal outcomes and variables such as offense, health and mental health, life events in prison, and/or history of child-hood abuse [4, 16–18]. While this evidence has advanced the knowledge in the field, there are several important limitations that warrant further research. First, for other regions in Spain these findings might diverge. The 2020 suicide rates per authority differ with 14.0 suicides per 10,000 incarcerated individuals in Catalonia (northeast of Spain) and 10.8 in the rest of Spain [2]. Indeed, Spain has two authorities in charge of the prison services: the *Catalan Secretary of Penitentiary Services, Rehabilitation and Juvenile Justice of the Department of* *Justice and Interior* (covering 9 prison facilities in Catalonia) and the *General Directorate of Penitentiary Institutions of the Ministry of Interior* (covering 69 prison facilities in the rest of Spain) [19]. Second, most of the evidence to date has focused on examining risk factors including clinical, criminological variables, but few institutional variables have been explored among Spanish incarcerated individuals. Studies in the Murcia region (southeast Spain) found that near-lethal suicide attempts were associated with work status in prison, solitary confinement and disciplinary infraction [17], but not with perceived social support [16]. Nonetheless, little is known about the relationship between prison regime (e.g. security level) and suicide risk and there is limited evidence on how supportive prison-level resources such as education programs and mental health care services may contribute to reducing suicide risk in Spain. Finally, most Spanish studies focused on suicide attempts whereas different suicidal outcomes have been associated with other factors [20]; hence, a further examination of the factors associated with suicide risk may be relevant since they will provide a richer understanding of key protective factors for suicide in Spanish prison populations. Especially a further exploration of the modifiable prison-related factors that are subject to public health and policy change may give insights for future effective suicide prevention programs.

In this context, the current study aimed to estimate the prevalence and explore the associations between sociodemographic, clinical, criminal, and prison-level variables and suicide risk in a sample of Spanish male incarcerated individuals.

## Methods

### Sample and study design

Data from the PRECA study was analyzed. PRECA [Spanish acronym for Prevalence of Mental Disorders in Prison] was an epidemiological study on mental disorders in prison facilities carried out between 2007 and 2008 in 3 Spanish regions (Aragon, Catalonia and Madrid). A stratified random sampling procedure was used based on the identification numbers provided by the State and Catalan Administration. To be eligible for this study, participants had to be male incarcerated individuals, aged from 18 to 75 years old, sentenced to imprisonment and with mental capacity to grant informed consent. Exclusion criteria were being on remand, being resident on a prison psychiatric ward, being about to be transferred to another prison or imminent release (free within 6 months), and having insufficient knowledge of the Spanish language.

A total of 783 convicted incarcerated individuals were eligible for the study and were invited to participate. Of them, 707 (90.3%) accepted to participate while 76 refused. From the 707 participating incarcerated individuals, 235 were located in Madrid, 222 in Catalonia and 250 in Aragon. The number of individuals incarcerated in these regions represents 28.8% of all incarcerated individuals in Spain [21].

Details of the survey methodology have been published elsewhere [19, 22]. Briefly, face to face interviews were undertaken in 5 Spanish prisons (i.e., Quatre Camins (Barcelona), Ponent (Lleida), Zuera (Zaragoza), Alcalá-Meco (Madrid) and Navalcarnero (Madrid). Clinical interviews were performed by six psychologists (2 per region) with clinical and/or research experience. To ensure interrater reliability, the interviewers received a 3‐day training period before data collection started. Sociodemographic, clinical and criminological data were collected via face-to face interviews and criminological data were confirmed using penitentiary records. Complementary clinical data (i.e. personality disorder, suicide risk) were collected with self-administrative questionnaires, which participants completed during the interview sessions.

Ethical approval was provided by the Sant Joan de Deu and Gol I Gorina Clinical Research Ethics Committees (Ref: 5/06; March 06) and was authorized by the relevant prison administrations. Written informed consent was obtained from all participants.

### Measures

#### Suicide risk

The Spanish version of the Plutchik suicide risk scale was used to assess the risk of suicide [23, 24]. It is a self-administered 15-item scale designed to discriminate individuals who are at risk of suicide from those who are not. The scale assesses, among others, previous suicide attempts, intensity of current suicidal ideation, feelings of depression and hopelessness. Each item includes a dichotomous response (yes vs no) and it is scored by giving a value of 1 to all affirmative answers and 0 to negative answers. The cut-off point for considering suicide risk is 6 points (Sensitivity 74% and Specificity 95%) [23, 24]. Of note, items 13 and 15 of the Plutchik scale respectively assess a lifetime prevalence of suicidal thoughts (yes/no) and history of suicide attempts (yes/no).

#### Sociodemographic variables

Sociodemographic variables included nationality (Spanish/Non-Spanish), age (categorized in 4 age-groups, 18–29, 30–39, 40–49 and > 50 years), educational level (no formal education, primary education, secondary education, university and other formation), current marital status (single/separated/divorced/widowed vs married) and prior working status (employed vs unemployed).

#### Criminological variables

Criminological variables included type of offense (violent offense/non-violent offense), recidivism (first time/re-offending), time until end of sentence (categorized as < 1, 2–3, 4–6, 7–9, and ≥ 10 years). Violent offenses were (attempted) homicide, murder, sexual offense, physical assault, domestic violence, violent robbery or robbery with intimidation, arson and, threats and coercion; non-violent offenses included robbery without violence or intimidation, sentence violation, and public health offenses. Individuals who were sentenced for both violent and non-violent offenses, were categorized as violent offense.

#### Clinical variables

Participants were asked about the presence of a family history of mental disorder. Information about their mental health symptoms and conditions (presence of life-time anxiety disorder, life-time affective disorder, life-time psychotic disorder and life-time substance disorder) was assessed using an adapted version of the Structured Clinical Interview for Diagnostic and Statistical Manual Axis I disorders (SCID-I; [25]).

The Spanish version of the International Personality Disorders Examination (IPDE) was used to evaluate the presence of a personality disorder [26]. Physical health variables included chronic morbidity and the following conditions were considered (Y/N): arthrosis, diabetes, low back chronic pain, cardiovascular diseases, irritable bowel, epilepsy, hepatitis type B/C, Human Immunodeficiency Virus (HIV) and Acquired Immune Deficiency Syndrome (AIDS). The answers to these questions were summed and the variable was operationalized as yes (≥ 1) or none.

Use of prison health services was operationalized as contact with a mental health specialist (yes/no) and medication treatment (yes/no), both with respect to the last 12 months.

#### Prison-related variables

Prison-related variables included whether the incarcerated individuals have been in solitary confinement (yes/no) during their time in prison, participation in workshops or training courses in the last 12 months (yes/no) and prison regime (first, second, and third grade). Workshops and training courses included training at different education levels (primary, secondary and university) and in labor skills, and educational, sports or artistic workshops. Concerning prison regime, first grade is for incarcerated individuals regarded as dangerous; they reside in isolation in maximum security wards or institutions (corresponding to the English category A). Second grade is for the majority of incarcerated individuals, consisting of an ordinary prison regime in ordinary wings in closed prisons (equivalent to the English categories B and C). Third grade (comparable with the English category D) is for incarcerated individuals who are serving prison terms under open conditions or are approaching the end of their sentence. Third grade incarcerated individuals may be released on parole [27].

### Statistical analysis

The difference in suicide risk on the items of the Plutchick scale and by sample characteristics was tested by Chi-squared tests. Logistic regressions were performed to examine the association between each risk/protection factors (predictors) and suicide risk (outcome). First, we conducted bivariate associations between predictor variables and suicide risk using binary logistic regression analysis. Next, we assessed the association between each of the correlates with suicide risk while adjusting for all sociodemographic variables (nationality, age, prison center, education level, marital status, and prior working status). A missing value analysis was conducted, showing that variables contained few missing cases. Generally missing values for individual items were less than 1%. This was deemed ignorable thus, for all analyses list-wise deletion was used and no imputation techniques were conducted. Results from the regression analyses are presented as odds ratios (ORs) with 95% confidence intervals (CIs). The level of statistical significance was set at *P* < 0.05. Statistical analyses were performed with Stata 14.1 (Stata Corp LP, College station, Texas).

## Results

The final sample included all 707 participants (mean age 36.79 years ± 9.90 years). The prevalence of high suicide risk was 30.6%. Table 1 shows the sample scores on the items of the Plutchik scale and the comparison of those who are at risk of suicide (*n* = 216) and those who are not (*n* = 491). The prevalence for a lifetime history of suicidal thoughts (item 13) and suicide attempts (item 15) was 33.8% and 21.1%, respectively. On all items, there was a significant association with suicide risk; those with an elevated suicide risk significantly more often confirmed to: take drugs or sleeping pills, have trouble falling asleep, fear to lose control, have little interest in being with people, expect an unpleasant future, feel worthless, feel frustrated, have felt so angry that they might kill someone, have had suicidal thoughts, and have attempted suicide.

**Table 1:** Scores on Plutchik scale (by sample and suicide risk)

			**Suicide risk**		
**Items**		**Overall** **n (%)**	**No** **n (%)**	**Yes** **n (%)**	***p* value**
1. Do you take drugs such as aspirins or sleeping pills regularly? ^a^	Yes	242 (34.6)	102 (21.0)	140 (65.4)	< .001
	No	457 (65.4)	383 (79.0)	74 (34.6)	
2. Do you have trouble falling asleep? ^b^	Yes	274 (38.9)	122 (24.9)	152 (70.4)	< .001
	No	431 (61.1)	367 (75.1)	64 (29.6)	
3. Do you sometimes fear that you will lose control of yourself? ^a^	Yes	212 (30.2)	90 (18.5)	122 (56.7)	< .001
	No	490 (69.8)	397 (81.5)	93 (43.3)	
4. Do you have little interest in being with people? ^b^	Yes	203 (28.8)	87 (17.8)	116 (53.7)	< .001
	No	501 (71.2)	401 (82.2)	100 (46.3)	
5. Do you feel that your future will be more unpleasant than pleasant? ^a^	Yes	192 (27.4)	69 (14.2)	123 (56.9)	< .001
	No	509 (72.6)	416 (85.8)	93 (43.1)	
6. Do you ever feel that you are worthless? ^b^	Yes	192 (27.4)	96 (19.6)	157 (72.7)	< .001
	No	509 (72.6)	393 (80.4)	59 (27.3)	
7. Do you feel hopeless about your future? ^a^	Yes	65 (9.3)	12 (2.5)	53 (24.7)	< .001
	No	637 (90.7)	475 (97.5)	162 (75.3)	
8. Do you often feel so frustrated that you just want to lie down and quit struggling altogether? ^b^	Yes	307 (43.7)	129 (26.5)	178 (82.4)	< .001
	No	396 (56.3)	358 (73.5)	38 (17.6)	
9. Do you feel depressed now? ^b^	Yes	118 (16.7)	31 (6.3)	87 (40.3)	< .001
	No	587 (83.3)	458 (93.7)	129 (59.7)	
10. Are you separated, divorced, or widowed? ^b^	Yes	234 (33.2)	140 (28.7)	94 (43.5)	< .001
	No	470 (66.8)	348 (71.3)	122 (56.5)	
11. Has anyone in your family ever tried to commit suicide? ^b^	Yes	109 (15.5)	50 (10.2)	59 (27.3)	< .001
	No	596 (84.5)	439 (89.8)	157 (72.7)	
12. Have you ever been so angry that you felt you might kill someone? ^b^	Yes	226 (32.1)	100 (20.5)	126 (58.3)	< .001
	No	477 (67.9)	387 (79.5)	90 (41.7)	
13. Have you ever thought about committing suicide? ^b^	Yes	238 (33.8)	59 (12.1)	179 (82.9)	< .001
	No	467 (66.2)	430 (87.9)	37 (17.1)	
14. Have you ever told anyone you would commit suicide? ^b^	Yes	93 (13.2)	16 (3.3)	77 (35.6)	< .001
	No	612 (86.8)	473 (96.7)	139 (64.4)	
15. Have you ever tried to kill yourself? ^b^	Yes	149 (21.1)	22 (4.5)	127 (58.8)	< .001
	No	556 (78.9)	467 (95.5)	89 (41.2)	

^a^0.7–1.1% missing data, ^b^0.3–0.7% missing data

Table 2 presents sample characteristics and the comparison of sociodemographic, criminological, clinical, and prison-related characteristics by group (risk vs no suicide risk). A sensitivity analyses was conducted, replicating Table 2 for a history of suicidal thoughts and a history of suicide attempts (item 13 and 15 of the Plutchik scale; see Supplementary material). The outcomes were largely the same, except that education level was not significantly associated with a history of suicidal thoughts, and marital status not with a history of suicide attempts.

**Table 2:** Sample characteristics (overall and by suicide risk)

			**Suicide risk**		***p* value**
**Characteristics**		**Overall** **n (%)**	**No** **n (%)**	**Yes** **n (%)**	
***Sociodemographic variables***					
Nationality	Spanish	513 (72.6)	336 (68.4)	177 (81.9)	< 0.001
	Non-Spanish	194 (27.4)	155 (31.6)	39 (18.1)	
Age (years) ^a^	18–29	208 (29.6)	142 (29.1)	66 (30.7)	0.206
	30–39	257 (36.6)	171 (35.0)	86 (40.0)	
	40–49	162 (23.0)	115 (23.6)	47 (21.9)	
	≥ 50	76 (10.8)	60 (12.3)	16 (7.4)	
Prison center	Quatre Camins	125 (17.7)	89 (18.1)	36 (16.7)	0.077
	Ponent	97 (13.7)	73 (14.9)	24 (11.1)	
	Zuera	250 (35.4)	168 (34.2	82 (38.0)	
	Alcalá-Meco	110 (15.6)	84 (17.1)	26 (12.0)	
	Naval Carnero	125 (17.7)	77 (15.7)	48 (22.2)	
Education level	No formal education	37 (5.2)	26 (5.3)	11 (85.1)	0.023
	Primary	447 (63.2)	292 (59.5)	155 (71.8)	
	Secondary	186 (26.3)	144 (29.3)	42 (19.4)	
	University	35 (5.0)	28 (5.7)	7 (3.2)	
	Others	2 (0.3)	1 (0.2)	1 (0.5)	
Marital Status	Married	216 (30.6)	172 (35.0)	44 (20.4)	< 0.001
	Single/separated or divorced/ widowed	491 (69.5)	319 (65.0)	172 (79.6)	
Prior working status	Employed	441 (62.4)	329 (67.0)	112 (51.9)	< 0.001
	Unemployed	266 (37.6)	162 (33.0)	104 (48.1)	
***Criminological variables***					
Type of offense	Violent offense	437 (61.8)	276 (56.2%)	161 (74.5%)	< 0.001
	Non-violent offense	270 (38.2)	215 (43.8%)	55 (25.5%)	
Recidivism	First time in prison	324 (45.8)	249 (50.7%)	75 (34.7%)	< 0.001
	Recidivist	383 (54.2)	242 (49.3%)	141 (65.3%)	
Time till end of sentence ^a^	≤ 1 year	241 (35.8)	174 (35.5%)	67 (31.5%)	0.512
	2–3 years	166 (24.7)	117 (23.9%)	49 (23.0%)	
	4–6 years	167 (24.8)	113 (23.1%)	54 (25.4%)	
	7–9 years	64 (9.5)	46 (9.4%)	18 (8.5%)	
	≥ 10 years	65 (9.2)	40 (8.2%)	25 (11.7%)	
***Clinical variables***					
Family history of mental disorders ^b^	Yes	198 (29.8)	106 (22.7%)	92 (46.5%)	< 0.001
	No	467 (70.2)	361 (77.3%)	106 (53.5%)	
Life-time prevalence of mental disorder					
Anxiety disorder	Yes	320 (45.3)	171 (34.8%)	149 (69.0%)	< 0.001
	No	387 (54.7)	320 (65.2%)	67 (31.0%)	
Personality disorder	Yes	582 (82.3)	378 (77.0%)	204 (94.4%)	< 0.001
	No	125 (17.7)	113 (23.0%)	12 (5.6%)	
Affective disorder	Yes	290 (41.0)	135 (27.5%)	155 (71.8%)	< 0.001
	No	417 (59.0)	356 (72.5%)	61 (28.2%)	
Psychotic disorder	Yes	76 (10.8)	32 (6.5%)	44 (20.4%)	< 0.001
	No	631 (89.2)	459 (93.5%)	172 (79.6%)	
Substance use	Yes	539 (76.2)	337 (68.6%)	202 (93.5%)	< 0,001
	No	168 (23.8)	154 (31.4%)	14 (6.5%)	
Physical conditions	Yes	331 (46.8)	186 (37.9%)	145 (67.1%)	< 0,001
	No	376 (53.2)	305 (62.1%)	71 (32.9%)	
Contact with a mental health specialist (last 12 months)	Yes	302 (42.7)	185 (37.7%)	117 (54.2%)	< 0.001
	No	405 (57.3)	306 (62.3%)	99 (45.8%)	
Medication treatment (last 12 months)	Yes	301 (42.6)	141 (28.7%)	160 (74.1%)	< 0.001
	No	406 (57.4)	350 (71.3%)	56 (25.9%)	
***Prison-related variables***					
Prison Regime ^a^	1	15 (2.1)	13 (2.7%)	2 (0.9%)	0.342
	2	672 (95.7)	466 (95.3%)	206 (96.7%)	
	3	15 (2.1)	10 (2.0%)	5 (2.3%)	
Solitary confinement (last 12 months) ^c^	Yes	170 (50.2)	106 (50.2%)	64 (50.0%)	0.966
	No	169 (49.9)	105 (49.8%)	64 (50.0%)	
	No				
Workshop or training course participation (last 12 months)	Yes	602 (85.1)	429 (87.4%)	173 (80.1%)	0.012
	No	105 (14.9)	62 (12.6%)	43 (19.9%)	

^a^ ≤ 0.7% missing data, ^b^5.9% missing data, ^c^ Information on solitary confinement was only available for those who were sanctioned during the last 12 months (*n* = 339; 48%)

The bivariate differences between both groups are presented in Table 3. Of those significant, the odds ratios ranged from 1.90 (working status prior to incarceration) to 7.09 (medication treatment in the last 12 months) for positive associations and from 0.48 (nationality) to 0.58 (workshop or training course participation in the last 12 months) for negative associations. After adjusting for control variables, the odds ratios were generally attenuated. In regard to sociodemographic variables, being single, separated divorced or widowed (aOR = 1.83; 95%CI = 1.23, 2.73; *p* < 0.01) and being unemployed prior to incarceration (aOR = 1.60; 95%CI = 1.12, 2.23; *p* < 0.01) were positively associated with suicide risk. Conversely, being non-Spanish (aOR = 0.53; 95%CI = 0.34, 0.81; *p* < 0.01) and being 50 years old or older (aOR = 0.51; 95%CI = 0.26, 0.98; *p* < 0.05) were both associated with decreased risk of suicide. As for criminological variables, having committed a violent offense (aOR = 2.02; 95%CI = 1.38, 2.95; *p* < 0.001) and being a recidivist (aOR = 1.45; 95%CI = 1.00, 2.11; *p* < 0.05) both increased the odds of suicide risk. Likewise, all clinical variables were independently associated with an elevated risk of suicide. Incarcerated individuals with a family history of mental disorders (aOR = 2.74; 95%CI = 1.82, 4.14; *p* < 0.001), the presence of an anxiety (aOR = 3.93; 95%CI = 2.71, 5.71; *p* < 0.001), personality (aOR = 4.33; 95%CI = 2.30, 8.17; *p* < 0.001), affective (aOR = 7.30; 95%CI = 4.93, 10.80; *p* < 0.001), psychotic (aOR = 3.28; 95%CI = 1.95, 5.51; *p* < 0.001), or substance use disorder (aOR = 5.00; 95%CI = 2.73, 9.09; *p* < 0.001) and physical conditions (aOR = 3.24; 95%CI = 2.23, 4.70; *p* < 0.001) had a 3- to sevenfold more likelihood to report suicide risk than those without these clinical characteristics. Also, contact with a mental health specialist (aOR = 1.90; 95%CI = 1.31, 2.73; *p* < 0.001) and medication treatment (aOR = 6.73; 95%CI = 4.52, 10.00; *p* < 0.001) in the last 12 months were associated with increased odds of suicide risk. On the contrary, the prison-related variable “participation in a workshop or training course during the last 12 months” (aOR = 0.51; 95%CI = 1.60, 3.12; *p* < 0.01) decreased the odds of suicide risk.

**Table 3:** Correlates of suicide risk estimated by logistic regression analysis

**Characteristic**		**OR**^**(a)**^	**95%CI**	**aOR**^**(b)**^	**95%CI**
***Sociodemographic variables***					
Nationality	non-Spanish vs Spanish	0.48***	[0.32,0.70]	0.53**	[0.34,0.81]
Age	18–29	Ref		Ref	
	30–39	1.08	[0.73,1.59]	1.06	[0.70,1.60]
	40–49	0.88	[0.56,1.37]	0.76	[0.47,1.22]
	≥ 50	0.57*	[0.30,1.07]	0.51*	[0.26,0.98]
Prison center	Quatre camins	Ref		Ref	
	Ponent	0.81	[0.44,1.48]	0.90	[0.48,1.70]
	Zuera	1.20	[0.75,1.93]	1.32	[0.80,2.14]
	Alcalá-Meco	0.77	[0.42,1.37]	0.86	[0.46,1.58]
	Naval Carnero	1.54	[0.90,2.61]	1.46	[0.84,2.53]
Level of education	No formal education	Ref		Ref	
	Primary	1.25	[0.60,2.60]	1.10	[0.51,2.37]
	Secondary	0.69	[0.31,1.51]	0.77	[0.34,1.75]
	University	0.59	[0.20,1.75]	0.90	[0.29,2.80]
	Others	2.36	[0.13,41.27]	3.24	[0.17,59.67]
Marital status	single/separated or divorced/widowed vs married	2.10***	[1.44,3.08]	1.83**	[1.23,2.73]
Prior working status	unemployed vs employed	1.90***	[1.36,2.61]	1.60**	[1.12,2.23]
***Criminological variables***					
Type of offense	violent vs non-violent	2.28***	[1.60,3.25]	2.02***	[1.38, 2.95]
Recidivism	recidivist vs first offender	1.93*	[1.39,2.69]	1.45*	[1.00, 2.11]
Time till end of sentence	≤ 1 year	Ref		Ref	
	2–3 years	1.09	[0.70,1.68]	1.18	[0.75,1.87]
	4–6 years	1.24	[0.81,1.91]	1.35	[0.86,2.13]
	7–9 years	1.01	[0.55,1.88]	1.07	[0.57,2.04]
	≥ 10 years	1.53	[0.73,3.22]	1.86	[0.85,4.08]
***Clinical variables***					
Family history of mental disorders	yes vs no	2.96***	[2.08,4.21]	2.74***	[1.82,4.14]
Anxiety disorder	yes vs no	4.16***	[2.95,5.86]	3.93***	[2.71,5.71]
Personality disorder	yes vs no	5.08***	[2.73,9.43]	4.33***	[2.30,8.17]
Affective disorder	yes vs no	6.70***	[4.70,9.57]	7.30***	[4.93,10.80]
Psychotic disorder	yes vs no	3.67***	[2.25,5.98]	3.28***	[1.95,5.51]
Substance use	yes vs no	6.60***	[3.71,11.71]	5.00***	[2.73,9.09]
Physical conditions	yes vs no	3.35***	[2.39,4.70]	3.24***	[2.23,4.70]
Contact with a mental health specialist (last 12 months)	yes vs no	1.95***	[1.41,2.70]	1.90***	[1.31,2.73]
Medication treatment (last 12 months)	yes vs no	7.09***	[4.94,10.18]	6.73***	[4.52,10.00]
***Prison-related variables***					
Prison Regime	1	Ref		Ref	
	2	2.87	[0.64,12.85]	3.26	[0.69, 15.38]
	3	3.25	[0.52,20.37]	5.84	[0.86, 39.65]
Solitary confinement	yes vs no	1.00	[0.64,1.53]	0.81	[0.50,1.33]
Workshop or training course participation (last 12 months)	yes vs no	0.58*	[0.38,0.89]	0.51**	[0.32,0.82]

^*^ *p* < 0.05, ** *p* < 0.01, *** *p* < 0.001

(a) Crude odds ratio. Each row represents a bivariate adjusted model

(b) Adjusted OR. Adjustments included: nationality, age, prison center, education level, marital status, and prior working status

## Discussion

Prison suicide is a complex phenomenon that entails multiple determinants that have not yet fully been investigated in the context of the Spanish prison system. This study analyzed secondary data to provide insight on the correlates of suicide risk in a sample of Spanish incarcerated individuals. Concretely, our study analyzed the correlates of suicide risk with socio-demographic, clinical, criminogenic and amenable, prison-related variables. Considering that the presented data is cross-sectional, it is worth emphasizing that casual relationships can only be speculated upon.

The prevalence rates of suicide risk, ideation and attempt in our study (30.6%, 33.8% and 21.1%, respectively) are in the range of those found among male incarcerated individuals in Europe [5–8] and in line with those found in Andalusian prison facilities (namely, 33.5%, 34.2% and 22.5%) [4]. Nonetheless, they greatly exceed the numbers in the Spanish general population (suicide ideation and attempts were 4.4% and 1.5%, respectively) [28], which was also found in a study in Catalan prison facilities, confirming that the suicide rate was eightfold higher in incarcerated individuals than in the general population [29].

In terms of sociodemographic correlates, we found a reduced suicide risk for those incarcerated individuals who are non-nationals of the country. This is in line with previous findings [6, 11, 30, 31], where it has been suggested that this association is driven by differences in suicide rates observed among different backgrounds in the general population [32, 33]. With respect to age, incarcerated individuals from the oldest group (i.e., ≥ 50 years old) were less likely to report suicide risk. Although some studies found increased odds of suicidal outcomes among younger incarcerated individuals [34, 35], others reported an increased risk for the older counterparts [33, 36, 37]. These contradictory results might suggest distinct patterns of suicide risk between younger and older incarcerated individuals. For instance, Stoliker et al. (2020) [38] found that suicidal behaviors and thoughts may manifest differently between age groups, with younger incarcerated individuals reporting more suicidal attempts and older incarcerated individuals more suicidal ideation. Nonetheless, in accordance with other studies, no such association was found between age and suicidal outcomes in our sensitivity analysis [5, 13]. It might be that our data reflect a “selection” effect, meaning that the lethality of suicidal behaviors among older incarcerated individuals decrease the odds of suicide risk. Namely, compared to younger individuals, older individuals are more likely to die from suicide on the first attempt due to the use of more violent methods, fearlessness regarding death, and a lower chance of being rescued because of physical vulnerability [38, 39]. We found that compared to being married, those who were single, separated/divorced or widowed were at higher risk of suicide, which is supported by previous research [7]. Marriage has long been identified as an important predictor of lower suicide risk in the general population [40] and it may confer its protection via increases in emotional and social support [41, 42]. Finally, as underlined by previous research [43], we also found that unemployment prior to incarceration increased the risk of suicide in prison.

As for criminological variables, we found that being a convicted of a violent offence and being a recidivist were significant risk factors for increased suicide risk. The association between offenses with extensive physical harm (e.g. homicide, assault and sexual offenses) and suicide risk is consistent with previous research [44–46]. Potential contributing factors to this increased risk are diminished levels of impulse control [47], aggressive or violent behavior [46] and/or psychopathic traits (e.g. Factor 2 anti-social lifestyle) [48]. Similarly, these factors may be contributing to the association found between increased suicide risk and recidivism in our study and by others [14], meaning that those exhibiting aggressive or violent behavior are more likely to be incarcerated more than once. The association between having perpetrated a violent offence and suicide risk may be explained by mental problems and/or substance misuse. For instance, a study of more than 27,000 adult suicides, linked with national criminal, psychiatric, sociodemographic and cause-specific mortality registers of a 26-year period in Denmark showed that after adjusting for psychiatric and social risk factors, the relative risk of suicide and severity of violent offense was attenuated [46]. Overall, prevention efforts targeted at these vulnerable groups should be especially considered by the Spanish prison authorities.

In terms of correlates pertaining to the clinical domain, we found an increased risk of suicide for incarcerated individuals with a family history of mental disorders. This finding is perhaps unsurprising, since these relationships have been previously reported in the general population [49]. Physical conditions, and the presence of a mental disorder and more specifically, affective disorders (e.g., depression) and substance use were the most important risk factors for suicide risk. Thus, the present study reaffirms that public health goals within prisons should include the improvement of the detection, prevention and provision of proper treatment of mental disorders [50]. In line with a study in Andalusian prison services [4], we found a high prevalence of personality disorders in our sample (82%). Studies using diagnostic tools (e.g. IPDE) are known to report higher prevalences than those based on clinical diagnoses [51]; nonetheless, a cautious interpretation is warranted here because there might be an overlap between the diagnostic criteria and the reasons for being incarcerated (i.e. criminological variables). Especially antisocial personality disorder, the most prevalent personality disorder among male incarcerated individuals [52], is characterized by disregarding norms and rules, having a low threshold for aggression or violence, and being unable to profit from experience, collectively exhibit strong correlations with criminogenic variables [51]. With respect to the clinical services within prison, our results also showed that contact with a mental health specialist and treatment with medication, both in the last 12 months, were related to a higher risk of suicide. A likely explanation here is that incarcerated individuals with underlying psychopathologies closely related to higher suicide risk (e.g. aforementioned affective disorders) have more treatment needs; hence, due to their increased suicide risk they receive more intensive treatment. This is consistent with studies in other prison populations [13, 53, 54], meaning that comprehensive treatment and close monitoring of incarcerated individuals with depression or other affective disorders is indicated to reduce prison suicide [14].

With respect to prison-related, amenable factors our study gave some interesting insights. In contrast with earlier studies [36, 55, 56], solitary confinement was not associated with suicide risk in our population. It is possible that the duration of the solitary confinement rather than the confinement per se may be associated with suicide risk. Long-term solitary confinement involves lack of social contact, increases apathy and lethargy, and increases the risk of developing symptoms of mental illness [57], factors that could ultimately lead to increased suicide risk. A lack of meaningful activities has been considered a risk factor for prison suicides [58, 59]. Indeed, we found that participation in a workshop or training during the last 12 months was significantly associated with reduced suicide risk in our population. Educational training courses and workshops (e.g. sports, arts) may decrease suicide risk by offering an environment in which incarcerated individuals can achieve a temporary mental “escape” from the pressures of imprisonment, by strengthening social relationships with peers, or by increasing their perceptions of preparedness to be reintegrated into society and overall wellbeing. Others reported positive associations in this regard such as perceptions of autonomy, safety, and relationships with staff [12]. Still, more research is warranted to examine the extent and nature of daytime activities in relation to suicide risk in the Spanish prison population. Of note, we did not find a significant relationship between prison regime and suicide risk. A potential explanation for the lack of significance is that our sample may be not sufficiently powered to detect significant associations since a 95.7% of the sample pertains to prison level 2. Future research is needed to better determine the role of prison regimen on suicide risk.

Overall, present findings include a number of recommendations relevant to public health and clinical practice – especially in Spain. Multifactorial prevention programs are needed to reduce prison suicide among Spanish incarcerated individuals. As indicated by others [9, 60], clinical, criminal and prison-related factors should be addressed at a multidisciplinary level, through involvement of health care services, criminal justice and prison administration. Ideally this should start at admission and continue throughout the entire imprisonment period. In order to be effective, suicide prevention policies should include pro-active screening of suicide risk and serious mental health problems, prison staff qualified in suicide risk assessment and management, intensive monitoring and psychological treatment for incarcerated individuals at risk (including substance dependent incarcerated individuals) and, promotion of purposeful daily activities by providing sufficient opportunities for employment, or other meaningful activities aimed at personal improvement and well-being [61, 62]. Limited use of solitary confinement, increased social support, and adequate and safe living conditions for incarcerated individuals at risk, should also be addressed at policy-level [63]. Here it is noteworthy that in 2014 the Spanish Ministry of internal affairs established a Framework Program for Suicide Prevention. The program contains a protocol for all prison professionals both to detect personal or social situations that may pose a high risk of suicide and to apply the most appropriate measures to prevent self-harming behavior. One might expect that the introduction of these policy frameworks decreased the number of suicides in Spanish prison facilities. Comparison of suicide rates per 10.000 incarcerated individuals between 2008 and 2015 (after the introduction of the framework) do indeed show a small decrease from 9.6 to 7.8 in Catalonia and from 4.7 to 4.2 in the rest of Spain [64, 65]. On the other hand, most recent numbers corresponding with 2020 show an increase with 14.0 and 10.8 suicide rates per 10.000 incarcerated individuals for Catalonia and the rest of Spain, respectively [2]. Seen this increase in suicide rates, the paucity of recent research on suicide risk among the Spanish prison population is concerning. To the authors’ knowledge there are no recent data on suicidal outcomes in a large, national Spanish sample of incarcerated individuals. Both, the Catalan and State authority responsible for the prison services in Spain should invest in studies that analyze the program and its effectiveness in preventing suicide among Spanish prisoners. Ideally, research should be embedded in the implementation of strategies to reduce suicide risk among incarcerated individuals as an iterative process of improvement.

The current study should be considered in light of some limitations. First, the study is cross-sectional, meaning that no temporal order between predictor and outcome could be ascertained. It is therefore equally possible that factors (e.g. contact with a mental health specialist) are *consequences* of increased suicide risk. Second, the suicide risk scale used in this study measures the total suicide risk. Although our sensitivity analysis did not reveal big differences in findings, previous research has shown that the present findings might diverge in relation to suicide attempts or ideation [6, 66]*.* Third, suicide risk was assessed via a self-reported measure, and thus, social desirability biases may be present. Fourth, several prison-related factors of potential importance for suicide risk in prison could not be analyzed such as prison violence and victimization [6, 7], nor did we have information on psychosocial factors such as social support and social visits [11, 62], or clinical factors such as suicidal ideation or suicide attempt during prison time, and self-harm behavior [11]. Fifth, current results cannot be generalized to female incarcerated individuals since only males were included in the present study. Whereas women have more suicidal ideation and attempts, men more frequently die by suicide [67]. Women are known to show other patterns of suicidal behavior [68] and are especially at risk if they have poor social and family support, prior suicidal behavior, a history of psychiatric illness, and emotional problems [69]. Likewise, individuals on remand were excluded from our study whilst the first months of incarceration generally represent a critical risk period for suicidal behavior [70]. Compared to those who are sentenced, incarcerated individuals on remand differ with respect to the timing of suicidal behavior and the adaptation to stressors such as sudden separation from relatives, novel environment, repeated court visits and uncertainty regarding the future [47, 69, 71]. Sixth, the data used for this study were collected between 2007 and 2008. Apart from being a considerable limitation, it also provides the opportunity for time-frame comparisons. As mentioned above, suicide rates among Spanish incarcerated individuals remain among the highest of Europe and new studies on this phenomenon are urgently needed. We investigated suicide risk and the correlates of a limited number of variables (due to the secondary data use). However, prison suicide is a complex phenomenon and sociodemographic, criminological, clinical and psychosocial factors are likely to interact with prison-related factors [21, 72]. Future studies should include a wide range of both individual and prison-related factors to provide a better understanding of the amendable prison-related factors, in comparison with individual factors, in the prevention of prison suicide in Spain.

## Conclusion

Current findings identified several risk and protective factors within a comprehensive range of sociodemographic, criminological, clinical and prison-related determinants among Spanish incarcerated individuals. This information is primordial for preventing suicide and reducing the existing risk. Importantly, some of these determinants represent treatable and modifiable factors that are subject to public health and policy change and, thus, may guide effective suicide prevention programs within prison facilities in Spain. Strategies to reduce suicide risk among Spanish incarcerated individuals should in particular target those who are unmarried/unemployed, with violent offenses, recidivism, and mental illness, and should include the provision of meaningful activities.

### Supplementary Information

**Additional file 1:** **Table S4**. Sample characteristics by lifetime history of suicidal thoughts and attempts (item 13 and 15 Plutchik suicide risk scale).

## Data Availability

The de-identified participant data is available as from publication and upon reasonable request from the corresponding author as long as the main objective of the data sharing request is replicating the analysis and findings as reported in this paper (without investigator support), after approval of a proposal, and with a signed data access agreement.

## Abbreviations

PRECA: Prevalencias de trastornos mentales en cárceles [Prevalence of Mental Disorders in Prison]
SCID-I: Structured Clinical Interview for Diagnostic and Statistical Manual Axis I disorders
IPDE: International Personality Disorders Examination
HIV: Human Immunodeficiency Virus
AIDS: Acquired Immune Deficiency Syndrome
OR: Odds Ratio
CI: Confidence Intervals
aOR: Adjusted Odds Ratio
