# Supplementary material for: Suicide risk in male incarcerated individuals in Spain: clinical, criminological and prison-related correlates
Source: BMC Psychol. 2023 Sep 21;11:282. doi: 10.1186/s40359-023-01315-y (PMC10514969; doi:10.1186/s40359-023-01315-y)
Supplement: Supplementary file 1 — Additional file 1: Table S4. Sample characteristics by lifetime history of suicidal thoughts and attempts (item 13 and 15 Plutchik suicide risk scale). [file 40359_2023_1315_MOESM1_ESM.docx]

**Table 4** Sample characteristics by lifetime history of suicidal thoughts and attempts (item 13 and 15 Plutchik suicide risk scale)

|  |  | **Suicidal thoughts** | | | **Suicide attempt** | | |
| --- | --- | --- | --- | --- | --- | --- | --- |
| **Characteristics** |  | **No**  **n (%)** | **Yes**  **n (%)** | **p value** | **No**  **n (%)** | **Yes**  **n (%)** | **p value** |
| ***Sociodemographic variables*** | |  |  |  |  |  |  |
| Nationality ^a^ | Spanish | 188 (79.0%) | 325 (69.6%) | .008 | 127 (85.2%) | 386 (69.4%) | <.001 |
|  | Non-Spanish | 50 (21.0%) | 142 (30.4%) |  | 22 (14.8%) | 170 (30.6%) |  |
| Age (years) ^a^ | 18-29 | 69 (29.2%) | 139 (29.9%) | .380 | 40 (26.8%) | 168 (30.4%) | .183 |
|  | 30-39 | 95 (40.3%) | 160 (34.4%) |  | 64 (43.0%) | 191 (34.6%) |  |
|  | 40-49 | 51 (21.6%) | 111 (23.9%) |  | 34 (22.8%) | 128 (23.2%) |  |
|  | ≥50 | 21 (8.9%) | 55 (11.8%) |  | 11 (7.4%) | 65 (11.8%) |  |
| Prison center | Quatre Camins | 37 (15.5%) | 88 (18.8%) | .191 | 22 (14.8%) | 103 (18.5%) | .060 |
|  | Ponent | 28 (11.8%) | 67 (14.3%) |  | 13 (8.7%) | 82 (14.7%) |  |
|  | Zuera | 95 (39.9%) | 155 (33.2%) |  | 63 (42.3%) | 187 (33.6%) |  |
|  | Alcalá-Meco | 31 (13.0%) | 79 (16.9%) |  | 19 (12.8%) | 91 (16.4%) |  |
|  | Naval Carnero | 47 (19.7%) | 78 (16.7%) |  | 32 (21.5%) | 93 (16.7%) |  |
| Education level | No formal education | 18 (7.6%) | 17 (3.6%) | .069 | 12 (8.1%) | 23 (4.1%) | .018 |
|  | Primary | 157 (66.0%) | 290 (62.1%) |  | 104 (69.8%) | 343 (61.7%) |  |
|  | Secondary | 52 (21.8%) | 134 (28.7%) |  | 28 (18.8%) | 158 (28.4%) |  |
|  | University | 10 (4.2%) | 25 (5.4%) |  | 4 (2.7%) | 31 (5.6%) |  |
|  | Others | 1 (0.4%) | 1 (0.2%) |  | 1 (0.7%) | 1 (0.2%) |  |
| Marital Status | Married | 61 (25.6%) | 154 (33.0%) | .045 | 40 (26.8%) | 175 (31.5%) | .276 |
|  | Single/separated or divorced/ widowed | 177 (74.4%) | 313 (67.0%) |  | 109 (73.2%) | 381 (68.5%) |  |
| Prior working status | Employed | 135 (56.7%) | 304 (65.1%) | .030 | 77 (51.7%) | 362 (65.1%) | .003 |
|  | Unemployed | 103 (43.3%) | 163 (34.9%) |  | 72 (48.3%) | 194 (34.9%) |  |
| ***Criminological variables*** | |  |  |  |  |  |  |
| Type of offense | Violent offense | 171 (71.8%) | 265 (56.7%) | <.001 | 117 (78.5%) | 319 (57.4%) | <.001 |
|  | Non-violent offense | 67 (28.2%) | 202 (43.3%) |  | 32 (21.5%) | 237 (42.6%) |  |
| Recidivism | First time in prison | 89 (37.4%) | 233 (49.9%) | .002 | 45 (30.2%) | 277 (49.8%) | <.001 |
|  | Recidivist | 149 (62.6%) | 234 (50.1%) |  | 104 (69.8%) | 279 (50.2%) |  |
| Time till end of sentence ^b^ | ≤1 year | 81 (36.7%) | 159 (35.3%) | 0.634 | 52 (38.0%) | 188 (35.2%) | .745 |
|  | 2-3 years | 55 (24.9%) | 111 (24.7%) |  | 33 (24.1%) | 133 (24.9%) |  |
|  | 4-6 years | 48 (21.7%) | 119 (26.4%) |  | 29 (21.2%) | 138 (25.8%) |  |
|  | 7-9 years | 23 (10.4%) | 40 (8.9%) |  | 14 (10.2%) | 49 (9.2%) |  |
|  | ≥10 years | 14 (6.3%) | 21 (4.7%) |  | 9 (6.6%) | 26 (4.9%) |  |
| ***Clinical variables*** |  |  |  |  |  |  |  |
| Family history of mental disorders | Yes | 89 (40.1%) | 109 (24.7%) | <.001 | 81 (58.7%) | 385 (73.2%) | <.001 |
|  | No | 133 (59.9%) | 333 (75.3%) |  | 57 (41.3%) | 141 (26.8%) |  |
| Life-time prevalence of mental disorder | |  |  |  |  |  |  |
| Anxiety disorder | Yes | 154 (64.7%) | 166 (35.5%) | <.001 | 41 (27.5%) | 344 (61.9%) | <.001 |
|  | No | 84 (35.3%) | 301 (64.5%) |  | 108 (72.5%) | 212 (38.1%) |  |
| Personality disorder | Yes | 215 (90.3%) | 367 (78.6%) | <.001 | 139 (93.3%) | 443 (79.7%) | <.001 |
|  | No | 23 (9.7%) | 100 (21.4%) |  | 10 (6.7%) | 113 (20.3%) |  |
| Affective disorder | Yes | 165 (69.3%) | 125 (26.8%) | <.001 | 115 (77.2%) | 175 (31.5%) | <.001 |
|  | No | 73 (30.7%) | 342 (73.2%) |  | 34 (22.8%) | 381 (68.5%) |  |
| Psychotic disorder | Yes | 47 (19.7%) | 29 (6.2%) | <.001 | 34 (22.8%) | 42 (7.6%) | <.001 |
|  | No | 191 (80.3%) | 438 (93.8%) |  | 115 (77.2%) | 514 (92.4%) |  |
| Substance use | Yes | 217 (91.2%) | 322 (69.0%) | <.001 | 140 (94.0%) | 399 (71.8%) | <.001 |
|  | No | 21 (8.8%) | 145 (31.0%) |  | 9 (6.0%) | 157 (28.2%) |  |
| Physical conditions | Yes | 148 (62.2%) | 183 (39.2%) | <.001 | 106 (71.1%) | 225 (40.5%) | <.001 |
|  | No | 90 (37.8%) | 284 (60.8%) |  | 43 (28.9%) | 331 (59.5%) |  |
| Contact with a mental health specialist (last 12 months) | Yes | 124 (52.1%) | 178 (38.1%) | <.001 | 83 (55.7%) | 219 (39.4%) | <.001 |
|  | No | 114 (47.9%) | 289 (61.9%) |  | 66 (44.3%) | 337 (60.6%) |  |
| Medication treatment (last 12 months) | Yes | 156 (65.5%) | 145 (31.0%) | <.001 | 104 (69.8%) | 197 (35.4%) | <.001 |
|  | No | 82 (34.5%) | 322 (69.0%) |  | 45 (30.2%) | 359 (64.6%) |  |
| ***Prison-related variables*** | |  |  |  |  |  |  |
| Prison Regime ^a^ | 1 | 2 (0.8%) | 13 (2.8%) | .141 | 1 (0.7%) | 14 (2.5%) | .378 |
|  | 2 | 227 (96.2%) | 443 (95.5%) |  | 144 (97.3%) | 526 (95.3%) |  |
|  | 3 | 7 (3.0%) | 8 (1.7%) |  | 3 (2.0%) | 12 (2.2%) |  |
|  |  |  |  |  |  |  |  |
| Solitary confinement (last 12 months) ^c^ | Yes | 68 (49.6%) | 101 (50.2%) | .912 | 44 (50.6%) | 125 (49.8%) | .901 |
|  | No | 69 (50.4%) | 100 (49.8%) |  | 43 (49.4%) | 126 (50.2%) |  |
| Workshop or training course participation (last 12 months) | Yes | 192 (80.7%) | 409 (87.6%) | .014 | 112 (75.2%) | 489 (87.9%) | <.001 |
|  | No | 46 (19.3%) | 58 (12.4%) |  | 37 (24.8%) | 67 (12.1%) |  |

^a^ ≤ 0.7% missing data, ^b^ 5.9% missing data, ^c^ Information on solitary confinement was only available for those who were sanctioned during the last 12 months (n=339; 48%)
